# Basic Uses of Python for Research in Our Laboratory: Examples of Data Analysis, and Processing of Histological Samples Based on the Special Graduate Lecture for Postgraduate Students Presented on January 2025

**DOI:** 10.14789/ejmj.JMJ25-0015-R

**Published:** 2025-07-31

**Authors:** JUAN ALEJANDRO OLIVA TREJO

**Affiliations:** 1 Department of Anatomy and Life Structure, Graduate School of Medicine, Juntendo University, Tokyo, Japan; 1 Department of Anatomy and Life Structure, Graduate School of Medicine, Juntendo University, Tokyo, Japan

**Keywords:** Python programming, AI, data analysis, image processing, morphology

## Abstract

Python is one of the most popular programming languages to learn and use. It is ubiquitous in fields like data analysis, artificial intelligence, image processing, robotics, and web development. Its versatility and reliability make it ideal for major companies, including Tesla and Netflix. NASA even uses it to calibrate the James Webb Space Telescope. With the advent of artificial intelligence (AI), writing a Python program can be as simple as prompting an AI assistant for a solution. For research scientists, this lowered barrier could spark a generational change, transforming every research stage from idea conception to publishing. My interest in AI-assisted programming inspired my lecture on how Python can assist PhD students in their work. This lecture begins with basic terminology and presents three mini projects as first steps for learning Python. These projects cover text extraction and analysis, statistical data analysis, and virtually staining histological samples into digital image stacks. Small variations of these projects could lead to code that extracts reagents from manuscripts, creates stunning statistical graphs, and advances histopathological methods.

## The Python programming language

Guido van Rossum, a Dutch programmer, released Python’s first code in 1991. It remains in active development, with the latest release in April 2025^1)^. Python is a high-level, dynamically typed, interpreted, and garbage-collected language. High-level code is easily readable by humans, unlike low-level code, which is closer to what computers understand. Being dynamically typed, variables can be undeclared at runtime. As an interpreted language, it doesn’t require compilation before execution. As a garbage-collected language, programmers don’t need to manually manage memory. These features made Python beginner-friendly early on and, over time, very popular.

Well-known companies rely on Python code daily for production. Netflix uses it for decision- making and managing content delivery^2)^. Tesla, the automobile company with self-driving technology, uses it for rapid code iteration^3)^. In NASA, Python code is used in many projects^4^^-^^6)^ including calibrating the James Webb space telescope^7)^. This Python code, freely available on GitHub, lets users process and analyze telescope data. These examples show Python’s flexibility, versatility, and reliability, making it an excellent choice across industries.

Python’s rise in popularity as a top programming language has been documented by the TIOBE Index and the Stack Overflow yearly survey of programming languages. The TIOBE Index measures language popularity through search engine activity. It shows Python became the most popular programming language around mid-2023^8)^. Similarly, the 2024 Stack Overflow survey shows Python as the most popular language for people learning to program and the third most popular overall^9)^.

Traditionally, writing Python code required pro grammers to invest significant time in learning. Now, Python code can be generated by prompting an AI-powered chat assistant like Grok^10)^ or ChatGPT^11)^. The potential of AI-generated code is currently unknown, as AI assistants’ programming skills are rapidly evolving. This ability to easily generate code can impact all fields, including scientific research. By widely using code in their work, even scientists in small labs could create tools to quickly extract data from papers, segment thousands of microscope images with AI, or monitor physiological parameters of many patients in real time.

## Starting Python

Getting started with Python is easy for a first-time programmer. The Python Software Foundation^12)^, the institution overseeing the development and guidance of the programming language, offers a guide for installing Python on your computer^13)^. This method installs a Python interpreter, the core engine that runs Python code, Python’s standard library, a basic code editor called IDLE, a package manager called pip, documentation, and other tools. Following this method, users get the basic tools to write and run Python code, plus a package manager to extend Python’s capabilities by installing additional libraries^14)^.

There are also several online Python interpreters like Replit^15)^, Online Python^16)^, and others, where code is interpreted by online servers. Another option is installing a specialized integrated development environment (IDE), like Spyder^17)^ or PyCharm^18)^. IDEs help with writing code by offering a code editor and interpreter for running programs. They also provide tools for finding bugs and features like syntax highlighting—making code easier to read by coloring different elements, such as variables, in distinct shades—to improve the coding experience. Additionally, IDEs include project management features, a terminal to use pip or manage files, and tools for working with online code repositories.

**Figure 1:**
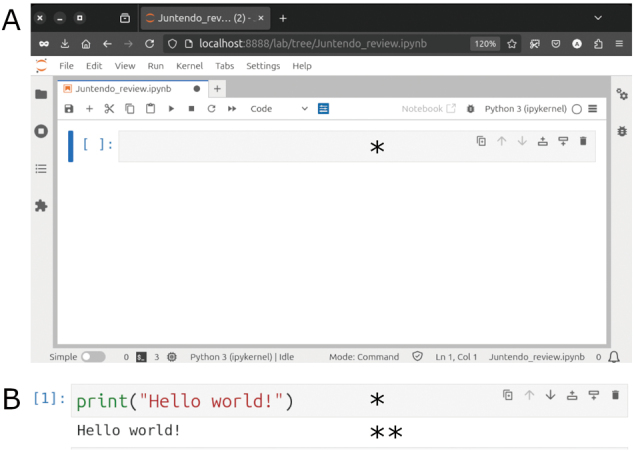
A) User interface of JupyterLab software running inside Firefox in the Ubuntu operating system. Asterisk indicates the cell where code is written. B) Python statement to display “Hello World!” message. One asterisk indicates code cell and two asterisks indicate Python output.

For this review, the JupyterLab Desktop standalone application is used^19)^. This application is great for beginners because it lets users mix code, text, images, and interactive visualizations and see the results instantly after running a cell. The files it produces, called notebooks, are quickly becoming a standard for writing, sharing, and inspecting code. JupyterLab runs in a web browser with a simple interface (Figure 1A). The code input space is called a cell, and code is executed by selecting the run command from the menu, clicking the “play” or triangle icon in the toolbar above the main working area, or pressing the shortcut keys Shift + Enter. All code for this review is referenced as notebook cell and it’s collected in a Jupyter notebook as supplementary data.

## Running Python code for the first time

A common first piece of code taught when learning programming is displaying “Hello world!” on the screen. In Python, this can be done by executing the following statement:

print (“Hello world!”)

A statement is a single line of code that performs an action when the interpreter runs it. This statement consists of the print function and an argument in parentheses. The print function outputs text or data, and the argument is the value passed to it—in this case, the text "Hello world!" (Notebook cell #1, Figure 1B).

A detailed explanation of all basic Python programming elements is limited due to space constraints, but key terminology is summarized in Table 1. With these basics, we'll explore three mini projects that highlight different fields, all useful for scientists to advance their work:

• Analyzing text and generating word clouds using Python.

• Statistical analysis and customization of plots.

• Converting histological images digitally into virtual Haematoxylin-Eosin (H&E) stains.

**Table 1:** Basic definitions of Python programming constructs used in this review

Python programming constructs	
Variable	A name that refers to an object or value that is stored in the computer’s memory. This value can be anything from a number to a name, to the whole text of a pdf file. It assigned using the “=” sign.
Function	Comprises a piece of code that executes a specific action.
Method	A function linked to an object or class.
Library	A collection of related functions, methods, modules, etc. that make coding efficient.
Conditional	Flow control element that allows elements of code to be evaluated based on whether a condition is true or false.
Loop	Flow control element that allows a portion of code to be executed as long as some condition holds, or for a determined number of cycles specified in the code.

### Mini project #1: Analyzing text and generating word clouds using Python

A word cloud visually represents the frequency of words in a text document. The more often a word appears, the larger its representation is in the cloud. Word clouds are often used to analyze trends in social media posts, speeches, or scientific papers, making key themes stand out. With only a few lines of codes, word clouds can be customized to make them more fun and visually appealing for presentations.

**Figure 2:**
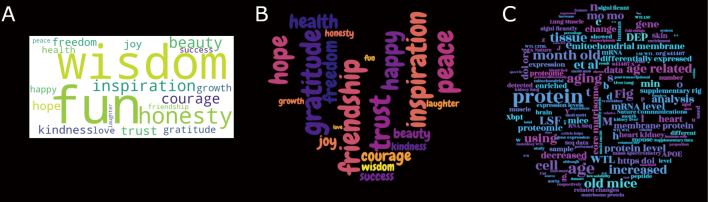
A) Simple word cloud without customization. B) Word cloud customized with a transparent background and heart shape. C) Word cloud generated from a PDF and customized with a transparent background and circle shape.

For this example, the code takes a group of words and generates a word cloud (Notebook cell 2). Here’s how it works: First, the needed libraries are imported: Word Cloud (for analyzing text to create the word cloud), Matplotlib (to generate the image of the word cloud), and random (to assign random values to each word in the example). The word cloud data consists of a list of 20 positive words. A random frequency is added to each word with some variability. The word cloud is created based on this data and finally displayed (Figure 2A).

The generated word cloud image is simple and not very aesthetically pleasing. To improve it, the word cloud can be customized by adding two new lines and editing one line of code (Notebook Cell 3, Figure 2B). These small changes enhance the word cloud by changing the font, selecting a new color palette with a transparent background, and replacing the default shape with a heart silhouette. These changes make the word cloud more engaging for presentations. Additional customizations can be made to match a project’s theme, or adjust the color palette to use your favorite colors for a personal flair.

Finally, the previous word cloud code will be updated to extract and analyze text from a published scientific manuscript. To do this, we add three new elements to the code: First, we import the PyPDF2 library and create a function to extract text from all pages of a PDF file. Second, we store the PDF file’s location in a variable on our computer. Third, we store the extracted text in a variable to create a word cloud. The previous code is updated to use a circular mask instead of a heart and a new font (Notebook Cell 4, Figure 2C).

This mini project shows how Python code can be used to analyze text and create customized outputs. By automatically analyzing text, we could also extract and list names of primary antibodies or reagents from a library of scientific papers. Automating this or other projects saves time by avoiding tedious manual extraction of information. For instance, code can be generated to summarize key findings from multiple papers for a literature review. This automation can also help track trends in research, like identifying common methods or topics across studies, making work more efficient.

### Mini project #2: Statistical analysis and customization of plots

The JetBrains Python Developers Survey 2023 (over 25,000 developers) identifies data analysis as Python’s most common use (47%). This includes applying statistical methods to dataset and to extract insights, highlighting its data-driven dominance^20)^. Libraries like Pandas and SciPy simplifying the processing of large datasets and performing statistical tests efficiently.

For this project, mock data are analyzed statistically using Python code (Notebook Cell 5). First, the stats module from the SciPy library (for science and math) is imported. Next mock data are stored in variables A and B. Then, using a single statement, the data in variables A and B are analyzed with a Student’s t-test. This outputs two results: t-statistic and p-value. For this example, we evaluate the p-value using a conditional statement against 0.05 (the chosen alpha value). The code concludes by displaying a message indicating whether there is a significant statistical difference (p-value < 0.05) or not (p-value > 0.05).

For a more realistic scenario, the next example uses Python code to evaluate statistical differences among three groups: A, B, and C. Each group has 100 data points, making it impractical to type each data point into a list, so a different approach for accessing the data will be shown. During the code execution, the data are checked for normality, evaluated statistically, and plotted into a graph.

The first step is to import the necessary libraries including Pandas for processing our code. Since the challenge is to automatically import hundreds of results without manually typing them we use Python code to extract data from a Microsoft Excel spreadsheet. First, the Excel file’s path is stored in a variable. Using the Pandas library the Excel file’s contents are extracted and the data are stored in a Pandas DataFrame for manipulation. The normality of each group is evaluated using the Shapiro-Wilk test. After confirming that the data are normally distributed, the groups are evaluated for statistical differences with a one-way ANOVA test. Just as the previous statistical test, a single Python statement suffices for analyzing the data. Finally, using a conditional loop, a post-hoc evaluation with Tukey’s test is evaluated and the output showing statistical difference is displayed on the screen (Notebook cell 6).

**Figure 3:**
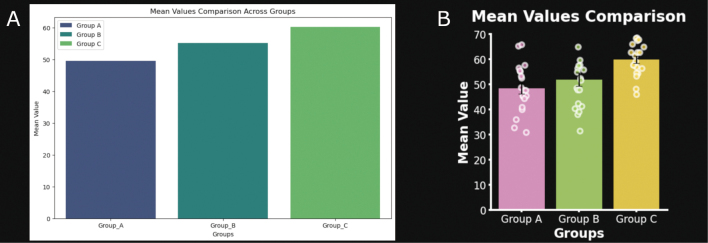
A) Bar plot generated with the seaborn library using the default settings. B) Customized bar plot. A dot plot showing the distribution of the data was overlayed on top of the bar plot. Other customizations include improved font settings, brighter colors, addition of error bars and a transparent background.

Plotting graphs in Python is straightforward using libraries like Seaborn or Matplotlib. For this example, Seaborn is used to generate a bar plot using the default options. We also use Matplotlib to add the graph title, label the x and y axes, and display the plot (Notebook cell 7, Figure 3A). Plots generated in Python can be customized extensively with minimal code by adding error bars and transparent backgrounds, using new fonts, changing color palettes, etc. It’s up to the user to create a graph that best meets their needs (Figure 3B).

For inspiration, websites like python-graph-gallery.com show graph plots and the Python code to reproduce them^21)^. Resources like these can motivate students to improve their Python skills and learn advanced data visualization techniques. For scientists, simple but outstanding visual representation of data in research is key for connecting science and understanding.

### Mini project #3: Digital histology

For the last mini project, Python code is used to transform fluorescent signals from histological samples into digitally stained images. This project may appeal to scientists who routinely analyze histological samples and may also be of broad interest as it highlights Python’s use in the laboratory.

The purpose of this mini project is to illustrate how Python code can be implemented in the laboratory. Therefore, some warnings are necessary: First, due to space constraints, there is only a brief description of the staining protocol and image stack acquisition. Additionally, this mini project requires the widely used software FIJI for preprocessing images.

This project is adapted from the PLOS ONE paper by Serafin R. et al., titled “False Color-Python: A Rapid Intensity-Leveling and Digital- Staining Package for Fluorescence-Based Slide-Free Digital Pathology”^22)^. The authors developed Python code and published the falsecolor module. Sample code was also prepared and published to the GitHub repository associated with the paper^23)^.

For this example, using Python code, a mouse glomerulus measuring around 65 µm is virtually stained. The alternative to this code would be to obtain 15 to 20 histological sections of a kidney, stain them individually, acquire images from the same region using a light microscope, and analyze them as a stack. This process would take at least 4-5 days. However, using the Python code workflow, results can be obtained on the same day or the next day.

The workflow is as follows: First, a 500 µm section of kidney is washed with PBS. Then, the cytoplasm is stained with fresh Eosin for 5 minutes on a shaker and washed with PBS. For the nucleus, DAPI is incubated for an hour. The sample is washed and optically cleared using two washes of 100% ethanol for 2 hours, followed by incubation in ethyl cinnamate. An image stack of the Eosin and DAPI fluorescence is acquired using a confocal microscope with UV and 568 nm filters. The image stack is then converted to an H5 file format using the BigStitcher plugin in FIJI with the default options^24)^.

**Figure 4:**
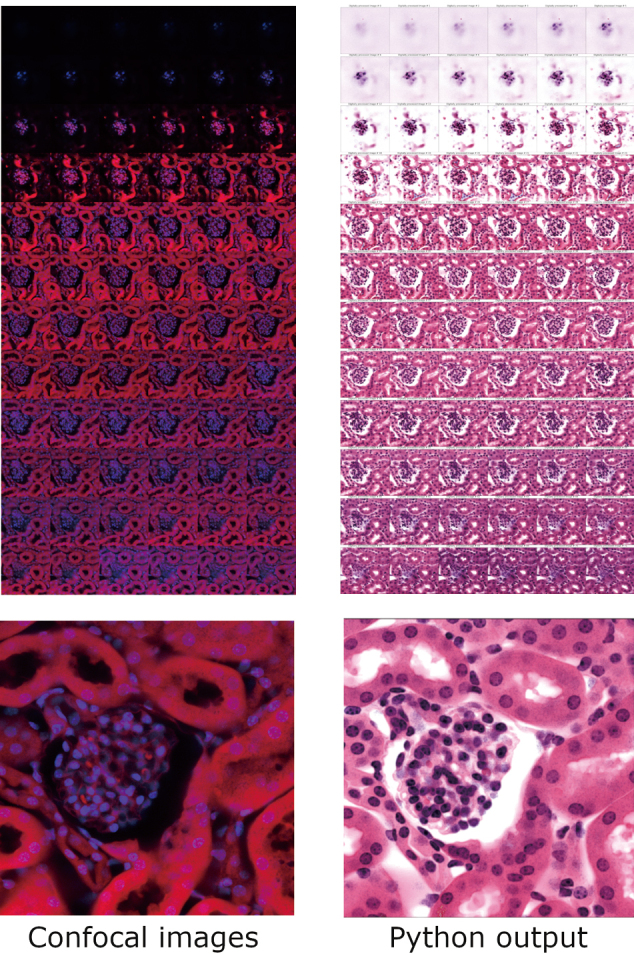
Sample images from a glomerulus image stack processed with FalseColor Python. Top left panel shows the image stack data acquired using a confocal microscope (red signal is for eosin autofluorescence; blue signal for nucleus marker DAPI). Top right panel shows the same image stack - after processing with Python code to simulate the Haematoxylin-Eosin (H&E) stain. Lower panels show magnified representative images from the confocal image stack (left) and the same image after processing with Python code (right).

Once the H5 file is obtained, Python code handles the rest: First, data from the nucleus and cytoplasm are extracted from the H5 file and stored in a variable. The images are then sharpened using the falsecolor module. Next, the color settings for emulating an H&E staining pattern are obtained from the module and applied to the images in the stack. Finally, the newly generated images are displayed using the ViewImage function of the falsecolor module (Notebook Cell 8, Figure 4).

## Conclusion

The mini projects shown here cover a small but diverse range of applications. They aim to highlight the usefulness and versatility of the Python programming language. From personal experience, Python adoption for laboratory work is low, while interest, as shown by the number of students attending the lecture, is very high. Leveraging the rise of AI-powered coding tools, alongside the creativity and needs of students, Python may become ubiquitous for this generation of researchers, as it is in the digital technology world.

## Author contributions

JAOT - conceptualized the review, developed the lecture content, drafted and edited the manuscript, designed the Python-based mini-projects (text extraction, data analysis, and virtual staining), and tested all associated code.

## Conflicts of interest statement

The author declare that there are no conflicts of interest.
